# Complete vaccination service utilization inequalities among children aged 12–23 months in Ethiopia: a multivariate decomposition analyses

**DOI:** 10.1186/s12939-020-01166-8

**Published:** 2020-05-12

**Authors:** Ayal Debie, Ayenew Molla Lakew, Koku Sisay Tamirat, Getasew Amare, Getayeneh Antehunegn Tesema

**Affiliations:** 1 Department of Health Systems and Policy, Institute of Public Health, College of Medicine and Health Sciences, University of Gondar, P.O. Box: 196, Gondar, Ethiopia; 2 Department of Epidemiology and Biostatistics, Institute of Public Health, College of Medicine and Health Sciences, University of Gondar, Gondar, Ethiopia

**Keywords:** Complete vaccination, Inequalities, Children, Ethiopia

## Abstract

**Background:**

Although World Health Organization works to make vaccination service available to everyone everywhere by 2030, majority of the world’s children have been unvaccinated and unprotected from vaccine-preventable diseases. In fact, evidences on factors contributing to changes in vaccination coverage across residential areas, wealth categories and over time have not been adequate. Therefore, this study aimed at investigating inequalities in vaccination status of children aged 12–23 months owing to variations in wealth status, residential areas and over time.

**Methods:**

Maternal and child health service data were extracted from the 2011 and 2016 Ethiopian Demographic and Health Survey datasets. Then, multivariate decomposition analysis was done to identify the major factors contributing to differences in the rate of vaccination utilization across residences and time variations. Similarly, a concentration index and curve were also done to identify the concentration of child vaccination status across wealth categories.

**Results:**

Among children aged 12–23 months, the prevalence of complete childhood vaccination status increased from 20.7% in rural to 49.2% in urban in 2011 and from 31.7% in rural to 66.8% in urban residences in 2016. The decomposition analyses indicated that 72% in 2011 and 70.5% in 2016 of the overall difference in vaccination status was due to differences in respondent characteristics. Of the changes due to the composition of respondent characteristics, such as antenatal care and place of delivery were the major contributors to the increase in complete childhood vaccination in 2011, while respondent characteristics such as wealth index, place of delivery and media exposure were the major contributors to the increase in 2016. Of the changes due to differences in coefficients, those of low wealth status in 2016 across residences significantly contributed to the differences in complete childhood vaccination. On top of that, from 2011 to 2016, there was a significant increment in complete childhood vaccination status and a 59.8% of the overall increment between the surveys was explained by the difference in composition of respondents. With regard to the change in composition, the differences in composition of ANC visit, wealth status, place of delivery, residence, maternal education and media exposure across the surveys were significant predictors for the increase in complete child vaccination over time. On the other hand, the wealth-related inequalities in the utilization of childhood vaccination status were the pro-rich distribution of health services with a concentration index of CI = 0.2479 (*P*-value < 0.0001) in 2011 and [CI = 0.1987; P-value < 0.0001] in 2016.

**Conclusion:**

A significant rural-urban differentials was observed in the probability of a child receiving the required childhood vaccines. Children in urban households were specifically more likely to have completed the required number of vaccines compared to the rural areas in both surveys. The effect of household wealth status on the probability of a child receiving the required number of vaccines are similar in the 2011 and 2016 surveys, and the vaccination status was high in households with high wealth status. The health policies aimed at reducing wealth related inequalities in childhood vaccination in Ethiopia need to adjust focus and increasingly target vulnerable children in rural areas. It is of great value to policy-makers to understand and design a compensation mechanism for the costs incurred by poor households. Special attention should also be given to rural communities through improving their access to the media. The findings highlight the importance of women empowerment, for example, through education to enhance childhood vaccination services in Ethiopia.

## Background

Vaccination is one of the disease prevention strategies for contagious childhood illnesses which saves 2–3 million deaths every year. Globally, about 86% of infants are vaccinated for vaccine-preventable diseases of pneumonia, Diphtheria, Hepatitis, Tetanus, Meningitis, Polio and Influenza. In 2018, about 86% of infants (116.3 million) received 3 doses of diphtheria-tetanus-pertussis (DTP3) vaccine worldwide to protect them against infectious diseases. About 129 countries reached at least 90% coverage of DTP3 vaccination in 2018, but 19.4 million children under the age of one year did not receive basic vaccines, and the proportion of the world’s children who receive the recommended vaccines has remained the same over the past few years [1, 2].

Even though one of the agenda of the World Health Organization (WHO) is to make vaccination services available to everyone everywhere by 2030, about 13.5 million children didn’t receive the initial dose of a vaccine due to lack of access to vaccination services. Besides, in the year 2018, about 60% of the world’s children were unvaccinated and unprotected from vaccine-preventable diseases [3]. Despite the availability of vaccines and governments in Sub-Saharan African (SSA) countries give high attention to childhood vaccination, child mortality related with vaccine-preventable diseases remains high [2]. Although the third dose of diphtheria, tetanus, and pertussis (DTP-3) vaccination was 86% in 2018, this national coverage hid the geographical and socio-economic inequalities of childhood vaccination [3].

In Ethiopia, infectious and communicable diseases account for 60–80% of the health problems [4], and a significant number of under-five mortality is due to vaccine-preventable diseases. The under 5 mortality rate stands at 67 per 1000 live births although the health sector transformation plan aims to achieve 30/1000 live births by 2020, and child immunization is held to be one of the indicators of progress [5]. The health policy of Ethiopia gives a strong emphasis to ensuring universal access to health care as indicated in the national Health Sector Development Program (HSDP) IV (2011–2015) [6]. An accelerated expansion of primary health care facilities, particularly health centers and health posts has been underway since 2003. As a result, each health post has two health extension workers, a total of 34,850 HEWs were trained and deployed so far, with the ratio to a population of 1:2301 that surpassed the HSDP III target of 1:2500 [6, 7]. The expansion was envisioned as the key strategy to deliver maternal, neonatal and child health interventions to the rural segments of the population [8]. The fifth National Health Accounts (NHA) in Ethiopia indicated that about 34% of the total health expenditure was household out-of-pocket spending [9]. It is imperative that such expansions contribute to health equity, and the 2010 World Health Organization report identified inefficient and inequitable use of resources as one of the factors that impede rapid movements towards the universal health coverage [10].

Residences and wealth status of the households are the two household level characteristics that have been examined extensively in earlier studies on inequalities in child health outcomes. The effects of these characteristics on child vaccination status are however ambiguous, reflecting large cross-country differences. For example, studies done in Pakistan [11], Nigeria [12] and India [13] findings indicated inequalities in vaccination coverage disfavoring children lived in households with lower wealth status. On the contrary, the findings in Brazil [14] indicated that lower vaccination rates among children from households with higher wealth status. These findings may reflect differential opportunity of time costs that mothers or caregivers face in making regular visits to immunization centers [13, 15]. Few studies have reported significant rural-urban disparities in child vaccination across developing countries and the spatial inequalities in availability and access to health facilities and information are pervasive, rural communities remain underserved in child vaccination in Ethiopia [16], Nigeria [17] and Pakistan [11]. On the other hand, studies have reported lower vaccination rates among children in urban areas, especially slum and informal settlements in Kampala [18] and Nairobi [19]. Such differences may arise from differences in development strategies as well as the definition of rural and urban areas. The existence of significant differences across countries and localities in the sources of inequalities in childhood vaccinations due to variations in structural, cultural and institutional settings [20]. The presence of such differences require that further studies are undertaken to identify the country-specific extent and sources of inequalities in childhood vaccination coverage since there was no documented evidences on factors contributing to differences in vaccination coverage across residences and wealth categories in Ethiopia.

However, studies done earlier for example Lakew et al. [21] considered only residence and wealth status as independent variables to assess childhood complete childhood vaccination status.

The finding of this study could critically inform policy makers to narrow the disparity in complete childhood vaccination status across residences and wealth status of households. Therefore, the study aimed to investigate factors contributing to the change in complete childhood vaccination service utilization across residential areas, time and the variation in the concentration of childhood vaccination status across wealth categories.

### Conceptual framework

**Fig. 1:**
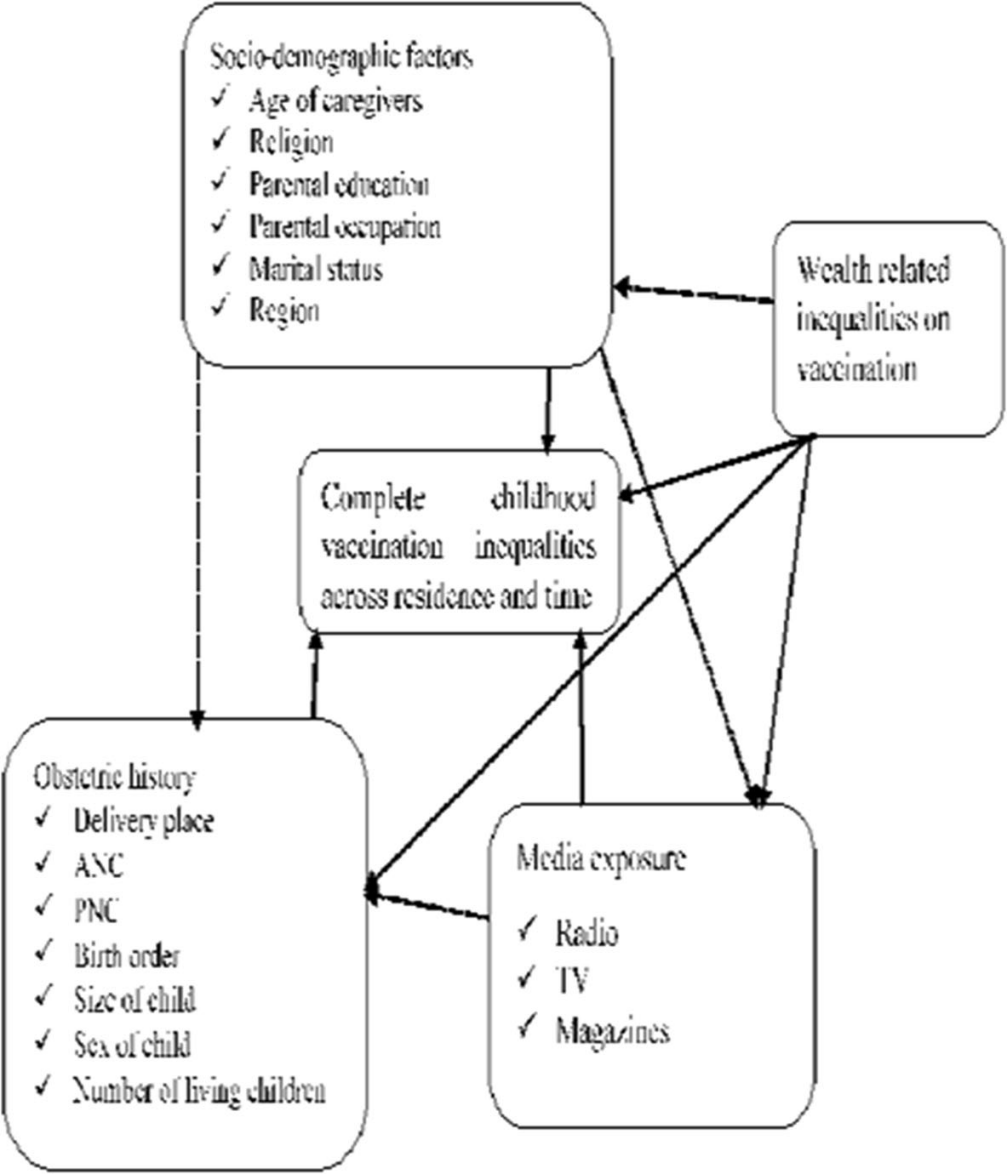
Conceptual framework on the factors affecting complete childhood vaccination inequalities across residential areas and over time in Ethiopia, EDHS 2011 and 2016

Conceptual framework is a generative framework that reflects the thinking of the entire research process. The diagram is created to clearly define the constructs or variables of complete childhood vaccination service inequalities and show their relationships using arrows. The figure presented that factors, such as socio-demographic characteristics, household wealth status, obstetric characteristics, and respondent’s media exposure could affect complete childhood vaccination service utilization inequalities across residences and over time. It is also displayed the interaction of different independent variables across one another, for example, the socio-demographic characteristics of the participants could affect childhood vaccination inequalities, obstetric history and media exposure of the respondents. Moreover, the framework illustrated the interaction of complete childhood vaccination service utilization inequalities across wealth categories (Fig. 1).

## Methods and materials

### Study settings and socio-economic characteristics

The 2011 and 2016 Ethiopian Demographic and Health Surveys (EDHS) datasets were available publicly via www.measuredhs.com. The EDHS data are nationally representative household surveys conducted at every 5-year intervals in the nine national regional states and the two city administrations. Maternal and child health service data were extracted from the data sets. A DHS report is considered as an important source of information for monitoring population health indicators and vital statistics in middle and low-income countries [22, 23]. The 2011 and 2016 EDHS Wealth Index (WI) was considered as a living standard measure for each respective year that used the Principal Component Analyses (PCA) for variables constructed as measures of socioeconomic variables. These variables included in the PCA were ownership of durable assets, like radios, cars, refrigerators, TV sets, motorcycles, and bicycles; housing characteristics, such as number of rooms for sleeping and building materials (walls, floors and roofs); access to utilities and infrastructures, like electricity supply, source of drinking water, and sanitation facilities.

### Measurements

Child vaccination inequities assess the child’s vaccination service disparities with respect to wealth index and residence of the mother. A complete vaccinated child was defined as a child who received all the recommended vaccines (one dose of BCG, three doses of pentavalent, Pneumococcal Conjugate (PCV), and Oral Polio Vaccines (OPV), two doses of Rota vaccine and one dose of measles) before its first birth day [24, 25]. Otherwise, a child who did not receive at least one dose of the recommended vaccines was considered as not full vaccinated [24–26] except PCV and Rota vaccines in 2011.

## Data management and analyses

Studies examining socioeconomic inequalities in health outcomes have applied the concentration index and concentration graph approaches [27, 28]. The study therefore used a concentration curve to identify whether socioeconomic inequality in some health variable exists and to examine whether it is more pronounced at one point than another. Besides, the study also used a concentration index [29, 30] to quantify and compare the degree of socio-economic related inequality in a health variable [27, 31, 32].

These techniques though useful for measuring the health inequalities, it does not explain the pathways through which socioeconomic factors influence observed inequalities in health outcomes between groups. The technique however may not be useful for examining subgroup inequalities in health such as rural-urban differences [33]. To address this limitation, we used a multivariate decomposition non-linear model of analyses, and the model helps to partition a difference in the values between two groups into components owing to group differences in observed characteristics and to group differences in the estimated effects of those characteristics [34], and this provides more detail information about a certain health variable by assessing the relative contribution of specific covariates to the components.

### Decomposition analyses

#### Complete childhood vaccination across residential areas

A multivariate decomposition analysis of the changes in child vaccination service utilization with residence was employed to find the major factors contributing to the differences in the rate of vaccination utilization. The regression analyses were used to find the difference between urban and rural dwellers in vaccination utilization. The purpose of the multivariate decomposition analyses was to identify the source of differences in vaccination utilization between places of residence. The change in both the composition (endowment) of population and the effect of the characteristics (coefficient) between the surveys is important to know the source of the factors contributing to the differences in vaccination utilization among rural and urban residents. The multivariate decomposition analysis for non-linear response model utilizes the output of a logistic regression model since it is a binary outcome to parcel out the observed difference in vaccination utilization between residences. The difference in vaccination utilization between urban and rural residents can be attributed to compositional changes in population (difference in characteristics or endowment) and to changes in the effect of the explanatory variables (difference in coefficient) between the residences.

Logit based decomposition analysis technique was used for the analysis of factors contributing to the change in vaccination utilization to identify the sources of changes across residences. The changes in vaccination utilization across residences can be attributed to compositional changes between urban and rural residents and the effects of the selected explanatory variables. Hence, the observed difference in vaccination utilization between surveys is additively decomposed into characteristics (endowments) and coefficients (effects) of the characteristic’s components.

### Complete childhood vaccination over time

A multivariate decomposition analysis of the change in complete childhood vaccination was employed to answer the major contributing factors in the differences in the rates of complete childhood vaccination over the study periods of the EDHS 2011 and 2016. The purpose of multivariate decomposition analysis was to identify the factors for the change in complete vaccination in the last 5 years. Both changes in composition (Endowment) of the population and effect of the characteristics (Coefficient) between the surveys are important to assess the contributing factors for the increase in complete vaccination over time. The multivariate decomposition analysis for non-linear response models utilizes the output from a logistic regression model since it is a binary outcome to parcel out the observed difference in complete vaccination between the surveys into components. The differences in the rates of complete vaccination between the surveys can be attributed to the compositional change in population (difference in characteristics or endowment) and the change in the effect of explanatory variables (difference in coefficient) between the surveys.

Logit based decomposition analysis technique was used to identify the factors contributing to the change in childhood complete vaccination over the periods in the last 5 years. The changes of complete vaccination over time can be attributed to compositional changes between surveys and changes in the effects of the selected explanatory. Hence, the observed difference in childhood full vaccination between surveys is additively decomposed into characteristics (or endowments) component and a coefficient (or effects of characteristics) component.

For logistic regression, the Logit or log-odd of vaccination across residence/ over time is taken as:

$\mathrm{Logit}\ \left(\mathrm{A}\right)-\mathrm{Logit}\ \left(\mathrm{B}\right)=\mathrm{F}\;\left(\mathrm{XA}\upbeta \mathrm{A}\right)\hbox{-} \mathrm{F}\left(\mathrm{XB}\upbeta \mathrm{B}\right)=\frac{\;\left[\mathrm{F}\;\left(\mathrm{XA}\upbeta \mathrm{A}\right)\hbox{-} \mathrm{F}\;\left(\mathrm{XB}\upbeta \mathrm{A}\right)\right]}{\mathrm{E}}+\frac{\;\left[\mathrm{F}\;\left(\mathrm{XB}\upbeta \mathrm{A}\right)\hbox{-} \mathrm{F}\;\left(\mathrm{XB}\upbeta \mathrm{B}\right)\right]}{\mathrm{C}}\kern0.36em$The E component refers to the part of the differential owing to differences in endowments or characteristics. The C component refers to that part of the differential attributable to differences in coefficients or effects. The analyses was done using the recently developed multivariate decomposition for the non-linear model, a *mvdcmp* STATA command for the decomposition analysis of child vaccination [35].

### Concentration curve and index

The concentration curve displays the share of health accounted for by cumulative proportions of individuals in the population ranked from the poorest to the richest. The two key variables underlying the concentration curve are the health variable and the distribution of the subject of interest against the distribution of the variable capturing living standards [36]. The concentration curve plots the cumulative percentage of the health variable (y-axis) against the cumulative percentage of the population ranked by living standards beginning with the poorest and ending with the richest (x-axis). The concentration curve would be a 45^0^-line running from the bottom left-hand corner to the top right-hand corner indicated the absence of inequity. Furthermore, the concentration curve lying above and below the equality line (45^0^) indicated that the health variable is disproportionately concentrated between poor and rich, respectively [37]. The further concentration curve deviated from the diagonal line indicated the greater the degree of inequality. Moreover, the cumulative full vaccination coverage (y-axis) was plotted against the cumulative percentage of the population ranked by household wealth status beginning with the poorest and ending with the richest (x-axis). The concentration index was twice the area between the concentration curve and the diagonal line. The range of the concentration index was from − 1 to + 1. The sign of the concentration index indicates the direction of the relationship between the health variable (vaccination status) and the distribution of living standards (wealth status). Therefore, the magnitude of the concentration index reflects both the strength of the relationship and the degree of variability in the health variable [27, 38, 39]. Thus, the need for computing the point estimate of the Concentration Index (CI). An index is easily computed in a spreadsheet program using the following formula [40].

$$ \mathrm{C}\kern0.5em =\kern0.5em \left({\mathrm{P}}_1{\mathrm{L}}_2\hbox{-} {\mathrm{P}}_2{\mathrm{L}}_1\right)+\left({\mathrm{P}}_2{\mathrm{L}}_3-{\mathrm{P}}_3{\mathrm{L}}_2\right)+\dots +\left({\mathrm{P}}_{\mathrm{T}}{\hbox{-}}_1{\mathrm{L}}_{\mathrm{T}}\hbox{-} {\mathrm{P}}_{\mathrm{T}}{\mathrm{L}}_{\mathrm{T}}{\hbox{-}}_1\right) $$

where, P_T_ is the cumulative percentage of the sample ranked by economic status in group T and L_T_ is the corresponding concentration curve ordinate. T is the number of socioeconomic groups. The data were analyzed using STATA *conindex* commands. The CI is calculated as two times the area between the 45^0^ diagonal line and the concentration curve. As a result, CI > 0 showed that the health variable was disproportionately concentrated on the rich, and CI < 0 revealed that the health variable was disproportionately concentrated on the poor. Concentration index = 0 also indicated that the distribution was proportionate. The CI always lies in the range of (− 1, 1). Accordingly, CI = 1 displayed that the richest person had all of the health variables, whereas CI = − 1 indicated that the poorest person had all of the health variables [36].

## Results

### Socio-economic and demographic characteristics of the respondents

The EDHS 2011 indicated that 26.6% of the urban and 27.4% of the rural participants were below the age of 25 years. Accordingly, about 23.3% of the urban and 16.1% of the rural participants were residents of Addis Ababa and Oromia region, respectively. One-point 5 % of the rural and 27.2% of the urban respondents had secondary or above education. Moreover, the wealth status of about 35.2 and 5.9% of the rural participants were classified under the poorest and richest wealth category, respectively. The EDHS 2016 revealed that nearly a quarter (23.0%) of the urban and 29.4% of the rural participants were below the age of 25 years. In addition, about one-third of the rural and a quarter (25.6%) of the urban children were in the age range of 12–14 months. Accordingly, about 25% of the urban and 20% of the rural participants were residents of Addis Ababa and Oromia region, respectively. Furthermore, about 30% of the rural and more than 40% of the urban residents were Orthodox Christians. More than 90% of the participants in both urban and rural areas were married during the survey. Five percent (4.5%) of the rural and 43.5% of the urban respondents had secondary or above education. Moreover, the wealth status of about 40 and 12.1% of the rural participants were classified under the poorest and richest wealth category, respectively (Table 1).

**Table 1:** Percentage distribution of socio-demographic characteristics of the respondents based on the Ethiopian Demographic and Health Survey,2011 and 2016

Characteristics	Category	2011		2016	
		Rural (*n* = 1558) (%)	Urban (*n* = 331) (%)	Rural (*n* = 1507) (%)	Urban (*n* = 395) (%)
Age in years	15–24	27.4	26.6	29.4	23.0
	25–34	51.7	58.9	48.4	60.5
	35+	20.9	14.5	22.2	16.5
Age of children in months	12–14	28.2	26.3	32.7	25.6
	15–17	28.6	29.6	26.1	19.1
	18–20	23.2	13.0	22.2	25.1
	21–23	20.0	21.1	19.2	20.2
Region	Tigray	11.3	7.6	11.7	10.1
	Afar	9.4	5.7	9.7	5.3
	Amhara	13.4	4.5	11.0	3.0
	Oromia	16.1	10.0	18.1	3.3
	Somali	7.1	10.3	11.0	12.9
	Benshangul_Gumuz	9.9	4.2	10.0	1.5
	SNNP	15.2	3.9	13.6	5.3
	Gambela	7.9	7.3	6.4	9.4
	Harari	4.9	10.9	5.0	10.4
	Addis Ababa	0	23.3	0	25.1
	Dire Dawa	4.8	12.4	3.6	13.7
Religion	Orthodox	29.7	45.9	28.3	43.8
	Muslim	46.1	41.1	49.8	40.3
	Protestant	20.4	12.7	18.5	15.2
	Others	3.9	0.3	3.5	0.8
Current marital status	Unmarried	10.4	19.0	6.0	8.4
	Married	89.6	81.0	94.0	91.6
Respondents’ education	No education	74.8	33.8	69.6	28.6
	Primary	23.8	39.0	25.9	27.9
	Secondary or above	1.4	27.2	4.5	43.5
Education of partners	No education	56.6	17.8	57.5	24.6
	Primary	36.7	38.7	32.2	24.1
	Secondary or above	6.8	43.5	10.3	51.4
Wealth index	Poorest	35.2	4.2	38.3	20.3
	Poor	21.1	1.8	19.0	20.8
	Middle	19.5	13.0	16.6	21.0
	Rich	18.4	5.7	13.9	18.5
	Richest	5.9	75.2	12.1	19.5

### Obstetric characteristics

The EDHS 2011 showed that 39% of rural and over 80% of urban mothers had received at least one ANC service, and half (52.5%) of the rural and above 90% of the urban mothers had media exposure. Moreover, only 5% of the rural and over 60% of the urban mothers gave birth a health facility. On the other hand, the EDHS 2016 indicated that more than 60% of rural and over 90% of urban mothers had received at least one ANC service and a quarter (23.8%) of the rural and three-quarters (76.7%) of the urban mothers had media exposure on maternal and child related healthcare services. On the contrary, only 8.2% of the rural and 12.2% of the urban women received PNC services. Furthermore, one-third (33.0%) of the rural and more than 85% of the women gave birth at a health facility. Almost half of the children in both areas were female and approximately 30% of the children were so large in both urban and rural areas (Table 2).

**Table 2:** Distribution of maternal and child health service utilization across residence in Ethiopia, EDHS 2011 and 2016

Characteristics	Category	2011		2016	
		Rural (n = 1558) (%)	Urban (n = 331) (%)	Rural (n = 1507) (%)	Urban (n = 395) (%)
ANC	No	60.2	18.0	37.2	6.1
	Yes	39.8	82.0	62.8	93.9
No of ANC visits	1	4.8	4.4	7.9	4.3
	2	7.6	3.5	12.5	5.9
	3	11.0	23.9	26.7	14.6
	4+	76.6	68.2	52.9	75.2
PNC	No	96.5	86.0	91.8	87.8
	Yes	3.5	14.0	8.2	12.2
Media exposure	No	47.5	7.0	76.2	23.3
	Yes	52.5	93.0	23.8	76.7
Place of delivery	Home	95.3	38.0	67.0	13.4
	H/facility	4.7	62.0	33.0	86.6
No. of living children	1	16.5	34.3	18.1	33.7
	2	20.0	20.5	17.5	25.6
	3+	63.6	45.2	64.4	40.7
Sex of child	Male	52.3	51.2	48.6	50.1
	Female	47.7	48.8	51.4	49.9
Birth order	1	15.5	34.8	17.9	34.2
	2	16.5	18.0	53.1	55.4
	3+	68.1	47.2	29.0	10.4
Size of child	Large	31.3	29.6	30.9	31.4
	Average	34.7	48.1	38.4	47.1
	Small	34.0	22.3	30.7	21.5

### Childhood complete vaccination status based on residence

**Fig. 2:**
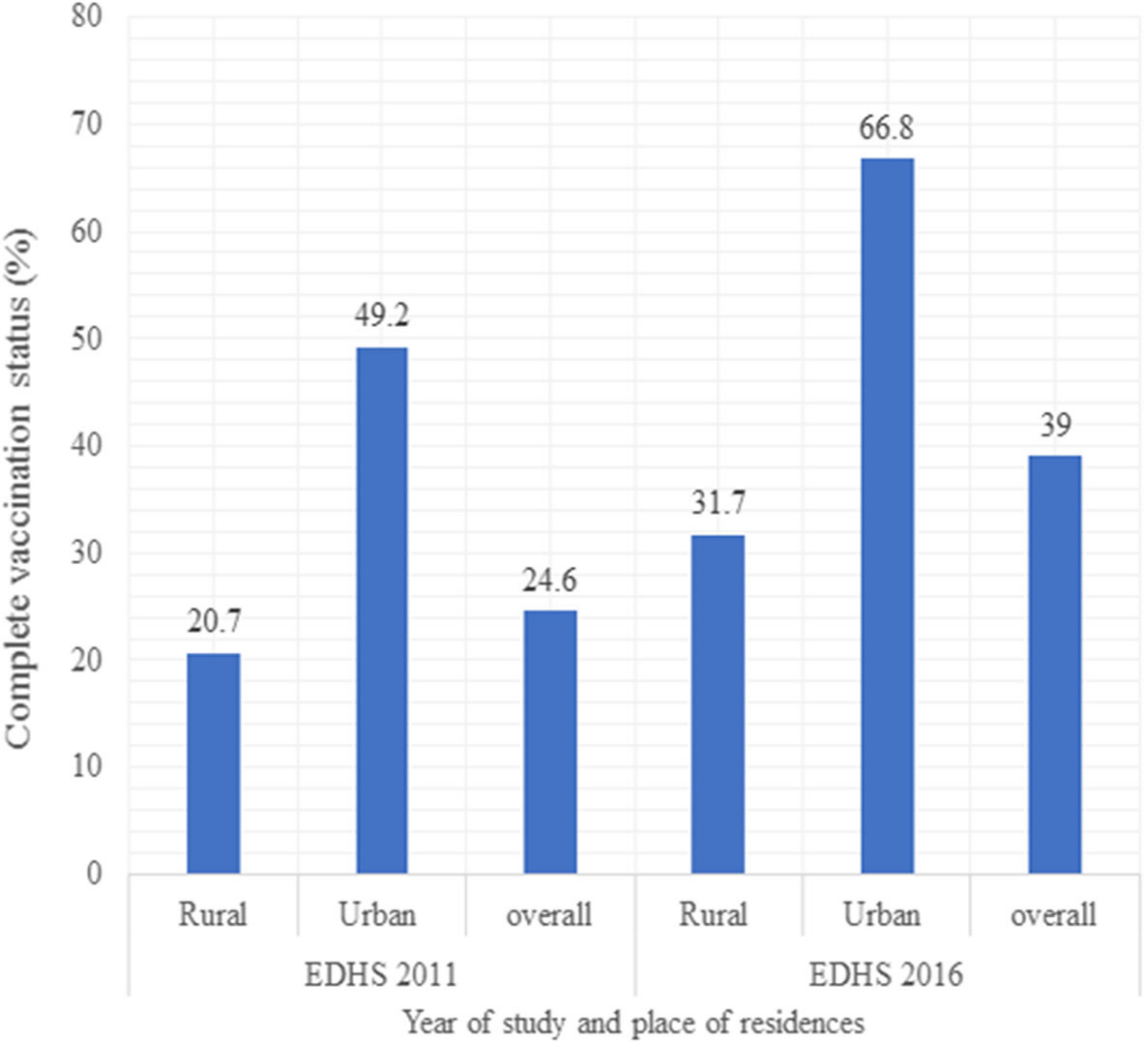
Distribution of complete childhood vaccination status among children aged 12–23 months across residential areas in Ethiopia, EDHS 2011 and 2016

The EDHS 2011 report indicated that the overall complete childhood vaccination in Ethiopia was 24.6% (95%CI: 22.7,26.6), while the 2016 found 39.0% (95%CI: 36.8, 41.2). Besides, complete childhood vaccination status across residences increased from 49.2% (95%CI: 43.1,55.2%) in urban to 20.7%(95%CI:18.8,22.7) in rural areas with the overall point difference of 28.5% in 2011. Similarly, the distribution of complete vaccinated children across residences in the EDHS 2016 report showed that there was a significant variation between urban 66.8% (95%CI:62.0,71.3) and rural 31.7%(95%CI: 29.3,34.0) areas, with the overall point of difference from rural to urban was 35.1% (Fig. 2).

### Residential distribution of childhood vaccination

This study indicated that the complete vaccination status of children increased among urban residents compared with rural ones. The EDHS 2011 showed that the complete childhood vaccination status of children in urban areas rose by 29.1, 38.2, 12.1 and 31.4% compared to rural settings, particularly when the mothers in the former residences used ANC, health facility delivery, and PNC services and had secondary school or above education, respectively. The EDHS 2016 indicated that the vaccination status of children in urban areas rose by 26.6, 34.1 and 28.5% compared to rural settings, particularly when the mothers in the former residences used ANC and PNC services and had secondary school or above education, respectively. Moreover, children’s vaccination status in the 2016 also increased by 40.3,36.7,26.0 and 45.5% among urban residents compared to rural areas, especially when the urban mothers had only one child each, were exposed to the media, delivered at health facilities and were in the richest wealth status, respectively (Table 3).

**Table 3:** Residential childhood complete vaccination distribution among children 12–23 months in Ethiopia Demographic and Health Survey in 2011 and 2016

Characteristics	2011			2016		
	Rural (n = 1558) (%)	Urban (n = 331) (%)	Point difference (Urban_ Rural)	Rural (n = 1507) (%)	Urban (n = 395) (%)	Point difference (Urban_ Rural)
Age in years						
15–24	19.9	58.0	38.1	32.3	67.0	34.7
25–34	25.3	53.3	28.0	31.6	65.7	34.1
35+	25.5	62.5	37.0	30.8	70.8	40.0
ANC						
No	16.5	17.9	1.4	14.1	37.5	23.4
Yes	34.5	63.6	29.1	42.1	68.7	26.6
PNC						
No	22.8	31.9	9.1	30.3	64.8	34.5
Yes	37.9	50.0	12.1	47.2	81.3	34.1
Delivery place						
Home	23.6	34.8	11.2	24.5	32.1	7.6
Health facility	28.9	67.1	38.2	46.2	72.2	26.0
No of living children						
1	22.3	62.8	40.5	34.9	75.2	40.3
2	24.9	66.7	41.8	34.5	70.3	35.8
3+	24.0	44.8	20.8	30.0	57.8	27.8
Media exposure						
No	20.9	28.3	7.4	29.5	39.1	9.6
Yes	27.2	60.4	33.2	38.6	75.3	36.7
Respondents’ education						
No education	22.8	41.1	18.3	26.7	45.1	18.4
Primary	26.7	60.5	33.8	41.8	70.9	29.1
Secondary/ above	36.4	67.8	31.4	50.0	78.5	28.5
Wealth index						
Poorest	16.9	21.4	4.5	16.5	42.5	26.0
Poor	18.9	16.7	−2.2	39.7	57.3	17.6
Middle	18.6	55.8	37.2	41.2	66.3	25.1
Rich	26.3	15.8	−10.5	42.4	83.6	41.2
Richest	38.3	61.9	23.9	41.5	87.0	45.5

### Decomposition analyses

#### Overall changes in vaccination status across residential areas

The overall decomposition analyses in the EDHS 2011 revealed that an increase in childhood complete vaccination in urban residents was due to the differences in characteristics (composition or endowment), and the change in ANC and institutional delivery service utilization were the significant predictors for the increase in childhood complete vaccination among urban compared with rural residents. On the other hand, the overall decomposition analyses in the EDHS 2016 showed that an increase in childhood complete vaccination status in urban residences was due to differences in characteristics (composition or endowment), and the marginal significance associated with the changes in the vaccination status of children was observed due to changes in the coefficient of characteristics.

#### Factors contributing to the changes in complete vaccination across residences

The decomposition analysis result in EDHS 2011 showed that about 72% of the increase in the childhood complete vaccination status attributed to the difference in composition of respondents across residences was explained by the difference in ANC and institutional delivery service utilization between urban and rural residents. An increased in ANC [β = 0.07; 95%CI: 0.03, 0.11] and institutional delivery [β = 0.13; 95%CI: 0.07, 0.20] service utilization among urban residents were significantly contributed for the differences in childhood vaccination. Over 60 and 40% of the differences in complete childhood vaccination were explained by the differences in ANC and institutional delivery service utilization across residential areas. The decomposition analyses result in the EDHS 2016 also showed that about 70.5% of the increase in the full vaccination status of children was attributed to the change in the composition of respondent’s characteristics explained by household wealth status, media exposure and place of delivery. Complete childhood vaccination was 66.8% among urban and 31.7% among rural children and the difference between the was significant. About 70.5% of the increase in full vaccination status of children in the urban areas was attributed to the difference in the effect of the change in composition of the characteristics of the respondents. The rate of change in child vaccination status due to the difference in composition of health facility delivery [β = 0.0854; 95%CI: 0.0012, 0.1696], richest wealth status [β = 0.0181; 95%CI: 0.0028, 0.03333], and media exposure [β = 0.0740;95%CI: 0.0011, 0.1469] were 24.3, 1.9 and 7.9%, respectively. On the other hand, of the changes due to differences in coefficients, the change in the effect of low wealth status [β = − 0.0283; 95%CI: − 0.0537, − 0.0023] across residences was significantly contributed about 8% of the decrease in childhood vaccination (Table 4).

**Table 4:** Residential decomposition change in complete vaccination status among children 12–23 months in Ethiopia, EDHS 2011 and 2016

Characteristics	Category	2011				2016			
		E		C		E		C	
		β(95%CI)	%	β(95%CI)	%	β(95%CI)	%	β(95%CI)	%
ANC	No								
	Yes	0.07 [0.03, 0.11] ^*^	62.6	0.03 [−0.006, 0.061]	29.8	0.0218 [− 0.0417, 0.0853]	6.20	−0.0910 [− 0.2039, 0.0219]	−25.86
PNC	No								
	Yes	0.01 [− 0.009, 0.03]	9.9	0.001[− 0.003, 0.005]	0.95	0.0020 [− 0.0049, 0.0090]	0.58	0.0008 [− 0.0125, 0.0141]	0.24
Delivery place	Home								
	H/facility	0.13 [0.07, 0.20] *	41.7	0.0009 [−0.03, 0.04]	0.3	0.0854 [0.0012, 0.1696] *	24.28	0.0227 [− 0.0227, 0.0681]	6.45
No. of living children	1								
	2	−0.0005 [− 0.005, 0.004]	− 0.4	− 0.002 [− 0.02, 0.02]	−1.9	0.0016 [− 0.0094, 0.0125]	0.44	−0.0027 [− 0.0262, 0.0209]	−0.76
	3+	0.003 [−0.002, 0.007]	2.6	−0.05 [− 0.11, 0.01]	−44.0	− 0.0006 [− 0.03141, 0.0302]	−0.17	− 0.0219 [− 0.1024, 0.0587]	−6.21
Media exposure	No								
	Yes	0.03 [−0.02, 0.07]	23.9	0.02 [− 0.02, 0.07]	19.8	0.0740 [0.0011, 0.1469] *	21.03	0.0276 [− 0.0009, 0.0561]	7.85
Education status	No- education								
	Primary	− 0.0005 [− 0.02, 0.02]	−0.46	0.001[− 0.018, 0.021]	1.2	0.0018 [− 0.0009, 0.0045]	0.50	0.0071 [− 0.0257, 0.0398]	2.01
	2^ry^ or above	−0.005 [− 0.022, 0.011]	−4.6	− 0.003[− 0.01,0.008]	−0.3	0.0328 [− 0.0245, 0.0901]	9.32	− 0.0006 [− 0.0077, 0.0066]	−0.16
Wealth index	Poorest								
	Poor	−0.03 [− 0.1, 0.04]	23.6	0.01 [− 0.04, 0.06]	10.4	0.00047 [− 0.00207, 0.00302]	0.13	− 0.0283 [− 0.0537, − 0.0023] *	−8.05
	Middle	− 0.002 [− 0.01,0.01]	−1.9	− 0.003 [− 0.04, 0.03]	−2.7	0.0018 [− 0.0056, 0.0092]	0.51	− 0.0244 [− 0.0484, − 0.0003] *	−6.92
	Rich	−0.008 [− 0.02, 0.007]	−7.2	0.007 [− 0.03, 0.04]	6.4	0.0089 [0.00020, 0.0176] *	2.53	0.0010 [− 0.0239, 0.0258]	0.27
	Richest	0.13 [− 0.01, 0.26]	112	0.003 [− 0.006, 0.01]	2.6	0.0181 [0.0028, 0.03333] *	5.14	0.0067 [−0.0173, 0.0307]	1.90

*Significant at *p*-value < 0.05, E: Difference due to Endowments; C: Difference due to Coefficient

#### Trends of childhood complete vaccination in Ethiopia from the EDHS 2011–2016

A significant increment in complete childhood vaccination was observed in Ethiopia from the EDHS 2011 to 2016. About 60% of the overall increment in complete childhood vaccination was explained by the difference in composition of respondents between the surveys, whereas the remaining 40.2% was due to difference in effect of characteristics (Coefficient). With regard to the change in composition, the differences in ANC service utilization [β = 0.03; 95%CI: 0.02, 0.04], health facility delivery [β = 0.03; 95%CI: 0.02, 0.05], urban residence [β = 0.003;95%CI: 0.0006, 0.006], secondary or above educated mothers [β = 0.006; 95CI: 0.0007, 0.012] and media exposure[β = 0.01; 95CI%: 0.02, 0.002] across the surveys were the significant predictors for the increase in complete childhood vaccination over time. Besides, the differences in the rate of complete vaccination due to the effect of characteristics (coefficient) was associated with the change in the effects of women primary education over time (the surveys) (Table 5).

**Table 5:** Change in complete vaccination status over time among children 12–23 months in Ethiopia, EDHS 2011 and 2016

Characteristics	Category	Difference due to characteristics(E)		Difference due to coefficient (C)	
		β(95%CI)	%	β(95%CI)	%
Residence	Rural				
	Urban	0.003 [0.0006, 0.006] *	3.5	0.009[−0.09, 0.027]	9.5
Maternal education	No education				
	Primary education	0.001 [0.0002,0.004] *	0.14	0.019 [0.0003, 0.038] *	20.2
	Secondary/ higher	0.006[0.0007, 0.012] *	6.5	0.004 [−0.003, 0.01]	4.05
Media exposure	No				
	Yes	0.01[0.02, 0.002] *	13.9	0.016 [−0.02, 0.05]	17.0
Place of delivery	Home				
	Health facility	0.03 [0.02, 0.05] *	35.7	−0.003 [−0.015, 0.009]	−3.1
Number of living children	1				
	2	−0.00004 [− 0.001, 0.001]	−0.04	− 0.01 [− 0.028, 0.008]	10.7
	3+	−0.00009[− 0.0002, 0.0001]	−0.1	0.014[− 0.03, 0.06]	14.5
ANC visit	No				
	Yes	0.03 [0.02, 0.04] *	33.9	−0.017 [−0.05, 0.012]	−18.5
Wealth status	Poorest				
	Poor	−0.003 [−0.005, 0.002]	−3.4	0.009 [−0.007, 0.026]	10.03
	Middle	−0.001 [− 0.002, 0.0005]	−1.1	−0.002 [− 0.017,0.013]	−2.0
	Rich	−0.005 [− 0.008, 0.030]	−5.6	0.0017 [− 0.013, 0.017]	1.8
	Richest	0.004 [−0.018, 0.0065]	4.4	−0.007 [− 0.03, 0.013]	−7.5

*Significant at *p*-value < 0.05

### Wealth related inequalities in complete childhood vaccination

#### Concentration index and curve

**Fig. 3:**
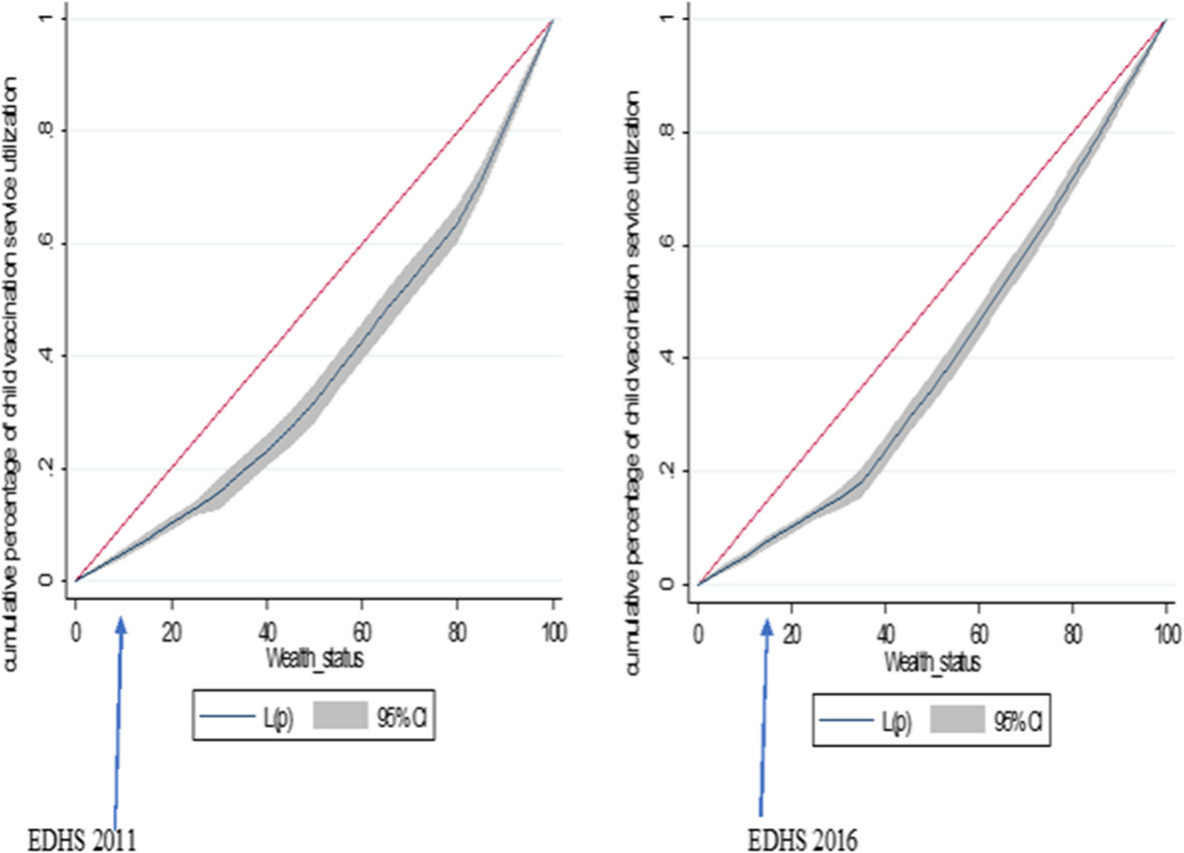
Concentration curves of wealth related inequalities for complete childhood vaccination status among children aged 12–23 months in Ethiopia, EDHS 2011 and 2016

The concentration index (CI) analyses showed that the wealth-related inequalities in the utilization of complete childhood vaccination was the pro-rich distribution of health services with CI = 0.2479 (*P*-value < 0.0001) and CI = 0.1987 (P-value < 0.0001) in the EDHS 2011 and 2016, respectively. The finding from the indices are in agreement with the results of the concentration curves in the two surveys. Similarly, the concentration curve indicated that the distribution of non-vaccinated children was concentrated in poor households in both surveys (Fig. 3).

## Discussion

This study was intended to examine complete childhood vaccination utilization inequalities and to identify the major contributing factors to the differences in the rate of complete vaccination across residences and over time. In relation with the trends, about 60% of the increase in complete childhood vaccination in the 2016 were attributed to the differences in the composition of respondent characteristics. Childhood vaccination status was increased more markedly in 2016 with a point difference of 14.4% compared to the 2011. This difference might be due to the variations in the increase in the uptake of maternal health services, such as ANC and institutional delivery. These services might enhance complete childhood vaccination service utilization. Moreover, the increasing in women empowerment, for example, female education might increase over time and this intern rises maternal decision-making power on child health services, such as vaccination.

The decomposition analyses also showed that about 72 and 70.5% of the increase in complete childhood vaccination status in urban residences were attributed to the differences in the composition of respondent characteristics in the EDHS 2011 and 2016, respectively. The complete childhood vaccination status of children aged 12–23 months increased more substantially in urban areas with an overall point difference of 28.5% in the EDHS 2011 and 35.1% in the 2016 compared to rural settings. This finding was supported by the studies done in Ethiopia [16], Nigeria [17] and Pakistan [11]. This might be attributed to differences in media exposure, service accessibility and level of knowledge about childhood vaccination. Another point is that most of the health facilities including private clinics and hospitals, are mostly situated in urban areas, increasing the availability and accessibility of vaccination services. This finding however differed from those of studies conducted in Ghana [41], India [42], Uganda [18] and Nairobi [43]. This disparity between different study areas might be due to the fact that the urban immunization settings have substantial barriers to childhood vaccination in spite of the physical access to health facilities and immunization centers, particularly in urban slums and informal settings. This finding is similar to that of a study conducted in Ghana. The difference could be due to the rapid pace of urbanization with ample growth of slums and informal settlements with inadequate healthcare facility coverages. Thus, the growing number of underserved children in the slums and informal settlements in the urban areas of Ghana have contributed to the observed low utilization of child vaccination in the country. In spite of the large spatial disparities in the access and utilization of health services, the child vaccination strategies in some developing countries appear to give more attention to rural areas through the Community-based Health Planning and Services (CHPS) programs. However, the urban-based CHPS has turned out to be much more challenging to implement as the population is more unstable and less well delineated [44].

In this study, over one-third (41.7%) and about one-fourth (24.3%) of the percentage change in childhood vaccination status in urban residence were due to differences in the composition of health facility delivery in 2011 and 2016, respectively. Similarly, over 60% of the percentage change in childhood vaccination status in urban residence were due to differences in the composition of ANC service utilization in 2011. This was supported by findings in Ethiopia [16], Nigeria [45], Pakistan [11] and Ghana [41]. The possible justification might that institutional delivery gives great opportunities for the administration of vaccines to the new born and facilities discussion, and better understandings of the immediate EPI schedules among mothers. The emergence of a significant relationship between places of delivery and the probability of full vaccination reflects the effects of improved access and utilization of health services on child health. In addition, in the last decades Ethiopia significantly expand and improved maternal and child health services, particularly in urban areas where people lived. Moreover, the healthcare system in Ethiopia might also lacks objective oriented strategy to monitor and evaluate healthcare services, particularly childhood vaccination service provision for those mothers who gave birth at home. Since most of rural mothers are non-educated, they did not properly understand the counseling services given about their child’s health during ANC visit. This might increase the magnitude of unvaccinated children in rural mothers attending ANC compared with the urban attendants.

The decomposition analyses also indicated that the change in child vaccination has a positive relation with maternal education in urban residences and about a 10% difference in 2016 was observed in child vaccination status between uneducated and secondary school or above educated mothers. Similarly, a 6.5% difference was also observed in childhood vaccination status between uneducated and secondary or above educated mothers from 2011 to 2016. This was supported by empirical literature which indicated that improving maternal education which was promoted globally as a mechanism to enhance child health outcomes in Zimbabwe [46], Nigeria [45, 47],and Ethiopia [16]. This might be due to the fact that an additional year of completed schooling of a mother exerts a positive effect on the probability of a child receiving the basic vaccinations. Maternal level of educational attainment enhances the access, reception of information and communication between health workers and mothers that can lead to better understanding of vaccination schedules and practices as indicated in Togo [48]. This is likely because low income is associated with both low levels of education as well as low literacy that could affect the awareness of mothers about disease prevention strategies, like childhood immunization [49]. The independent effect of education may be related to the increased awareness of existing programs.

Household’ wealth status was also one of the predictor variables of the change in the composition of childhood vaccination in urban residences. The decomposition analyses indicated that about 2% of the percentage change in child vaccination status in urban residences was due to the difference in the composition of household wealth status. Similarly, the concentration index analyses showed that the wealth-related inequalities in the utilization of full vaccination status of children was a pro-rich distribution of health services with a concentration index of 0.1987, and the concentration curve indicated that the distribution of fully vaccinated children was concentrated in the wealthiest households. This finding was supported by findings in Zimbabwe [46], Bangladesh [50], Nigeria [12] and India [13]. The possible justification might be that low wealth households might spend their time in income generating activities to prop up the household standards of living. As a result, poor people prioritize income rather than preventive health services, such as immunizations [51]. The other possible explanation for the wealth-related disparities in achieving full vaccination status may be found in the health seeking attitudes and practices of poor households and ignorance about vaccination associated with people who live at great distance from immunization center [51]. Besides, indirect costs required for travel to immunization centers or time lost away from income-generating activities make it difficult for the poorest households to avail themselves of services that exist in the community. This is also supported by the fact that poverty and marginalization are considered to be the major causes of inequalities in health [52]. The health policy of Ethiopia has no a compensation mechanism for the time and/ or transportation costs incurred by the poor households to give equitable and inclusive child healthcare to the target groups.

The decomposition analyses indicated that about 8% of the percentage change in child vaccination status in urban residences was due to variations in exposure to the media. This finding was in line with those of other similar studies in Ethiopia [16] and Sub-Saharan Africa (SSA) [53]. This might be due to the fact that the media can help to disseminate health information and facilitate behavioral changes. Therefore, in this study urban respondents had access to the media made arrangements for their children complete to their vaccination schedules.

### Limitations

The change in the effects of low wealth status across residences significantly contributed to the differences in childhood vaccination. As a result, the effects of the rates of change in vaccination difference due to the effects of coefficients might not well explained. Furthermore, the finding might lack clinical characteristics of the children and the wealth index of the households were calculated using durable assets rather than using household’s consumption since we used DHS data and these data sets did not have such information.

## Conclusion

The objective of this study was to investigate rural-urban inequalities in complete childhood vaccination coverage in Ethiopia between the EDHS 2011 and 2016. The health investments in the early child health have been found to be essential to later-life outcomes. Thus, socioeconomic disparities in health investments in early childhood may continue intergenerational poverty and inequalities as well as disparage the objective of equitable and inclusive growth.

The findings of this study reveal that significant rural-urban differentials were observed in the probability of a child receiving the required childhood vaccines. Children in urban households are specifically more likely to have completed the required number of vaccines compared to the rural areas in both surveys. The effects of maternal education suggest the importance of women empowerment to childhood vaccination. The concentration index and curve reveal the effects of household wealth status on the probability of a child receiving the required number of vaccines are similar in the 2011 and 2016 surveys. A high concentration of unvaccinated children was observed in the poorest households. The decomposition analysis of the rural-urban inequalities in childhood vaccination coverage reveals the existence of significant disparities in the probability of a child receiving the complete vaccination. The direction of the disparities in 2011 and 2016 surveys were also similar and exists in a rural disadvantage in complete childhood vaccination. Therefore, the health policies aimed at reducing wealth related inequalities in childhood vaccination in Ethiopia need to adjust focus and increasingly target vulnerable children in rural areas. It is of great value to policy-makers to understand and design a compensation mechanism for the costs incurred by poor households. Special attention should also be given to rural communities through improving their access to the media. The findings highlight the importance of women empowerment, for example, through education to enhance childhood vaccination services in Ethiopia. Moreover, community health workers had better make continuous and timely home visits to avoid missed opportunities for child vaccination by mothers delivering at home.

## Data Availability

The datasets used during the current study are available at Measure DHS website: http://www.measuredhs.com.

## Abbreviations

ANC: Antenatal Care
C: Composition
CC: Concentration Curve
CI: Concentration Index
DPT: Diphtheria, Pertussis, and Tetanus
E: Endowment
EA: Enumeration Area
EDHS: Ethiopia Demography Health Survey
EPI: Expanded program of immunization
PNC: Post Natal Care
SD: Standard Deviation
USD: US Dollar
VPD: Vaccine-Preventable Diseases
WHO: World Health Organization
